# Crystallization and 1.6 Å resolution crystal structure of an acylated GLP-1/GIP analogue peptide

**DOI:** 10.1107/S2053230X26001937

**Published:** 2026-03-17

**Authors:** Hamish M. Mitchell, Boguslaw Nocek, Emily J. Guinn, Jerry Y. Y. Heng

**Affiliations:** a Department of Chemical Engineering, Imperial College London, LondonSW7 2AZ, United Kingdom; b Discovery Chemistry Research and Technology, Lilly Corporate Center, Eli Lilly and Company, Indianapolis, IN46285, USA; c Synthetic Molecule Design and Development, Lilly Research Laboratories, Eli Lilly and Company, Indianapolis, IN46221, USA; d Institute for Molecular Science and Engineering, Imperial College London, LondonSW7 2AZ, United Kingdom; Centro Nacional de Biotecnología – CSIC, Spain

**Keywords:** GLP-1, peptides, crystallization, diabetes, obesity

## Abstract

With the meteoric rise in interest in GLP-1 and GIP analogue peptides in recent years, there is a drive for the use of alternative purification techniques to alleviate processing bottlenecks and reduce the cost of peptide manufacturing. However, a lack of reported crystal structures for this class of peptides has hindered molecular-scale understanding of GLP-1/GIP analogue peptide crystallization, particularly related to acylated peptides. This paper therefore reports what is believed to be the first crystal structure of a GLP-1 and GIP analogue lipopeptide. Crystals obtained using a microseed matrix-screening protocol diffracted to ≤1.6 Å resolution in space group *P*4_3_, with unit-cell parameters *a* = *b* = 64.66, *c* = 11.42 Å. Model building and the resultant structural analysis reveals that the predominantly helical peptide forms a uniquely porous spiral crystal structure composed of clockwise-ascending monomers in a square pattern, with aromatic C⋯H—π interactions around Phe22 forming the primary crystal contact between neighbouring square motifs.

## Abstract 2

The 1.6 Å resolution diffraction and subsequent structure solution of an acylated GLP-1/GIP analogue lipopeptide is reported. This represents the first published data on the crystal structure of an unbound GLP-1 and/or GIP analogue lipopeptide.

## 1. Introduction

Therapeutic peptides represent a unique and fast-growing sector of the global pharmaceutical market (Muttenthaler *et al.*, 2021 ▸) owing to their advantages over both small-molecule drugs (improved specificity and toxicity profiles; Rossino *et al.*, 2023 ▸) and biologics [simpler synthesis and development, lower immunogenicity, improved stability and storage (Wang *et al.*, 2022 ▸), as well as potential for oral delivery, as shown by therapeutics such as Rybelsus (Drucker, 2020 ▸)]. Currently, peptides are used to treat a variety of chronic diseases, such as diabetes, cancer and various autoimmune disorders (Muttenthaler *et al.*, 2021 ▸). Of these, the class of GLP-1 and GIP analogue peptides, used for the treatment of type 2 diabetes and obesity, have seen explosive growth in the past five years. Multiple blockbuster peptides such as semaglutide (Knudsen & Lau, 2019 ▸; sold as Ozempic, Wegovy and Rybelsus) have emerged in this class, and advancements in upstream manufacturing have enabled efficient synthesis techniques on industrially relevant scales (Frederick *et al.*, 2021 ▸). However, the separation and purification of these peptides are almost entirely achieved via chromatographic methods (Al Musaimi & Jaradat, 2024 ▸), which contribute significantly to the high costs of production and therefore drive up costs to patients. As a result, purification is often the bottleneck to peptide manufacturing (Isidro-Llobet *et al.*, 2019 ▸), and there is therefore a drive to explore alternative purification methodologies beyond chromatography.

Crystallization is one such alternative to chromatographic separations, and is already widely employed for the purification of small-molecule pharmaceuticals, offering high degrees of purification, lower operating costs and improved stability of the crystalline product (Roque *et al.*, 2020 ▸). In this context, crystallization is used as a preparative technique, where the isolation of a crystalline phase is sufficient and diffraction-quality crystals are not required. However, outside of insulin (dos Santos *et al.*, 2017 ▸), peptides are not commonly purified by crystallization. This is due in part to the prevalence of flexible sections of the peptide, coupled with the comparatively smaller size of peptides compared with macromolecules such as proteins. As a result, highly flexible portions of the peptide are inherently present on the surface of the peptide. The presence of highly entropic chains on the surface of the peptide has been identified as the most deleterious feature to crystallization (Price *et al.*, 2009 ▸), making peptides notoriously difficult to crystallize. This issue is exacerbated by the common addition of long, flexible conjugate groups (commonly fatty acids or polyethylene glycols) to the peptides in order to enhance their bioavailability (Wijesinghe *et al.*, 2022 ▸), which acts as a further detriment to crystallization. In the case of fatty acid-conjugated peptides, while limited exceptions do exist (Ho *et al.*, 2008 ▸), the resultant class of ‘lipopeptides’ is broadly perceived as a noncrystallizing class of molecule (Castelletto & Hamley, 2018 ▸; Pilz *et al.*, 2023 ▸).

Substantial research efforts have been dedicated to the crystallization of short peptides (Verma *et al.*, 2022 ▸; Verma, Bade *et al.*, 2023 ▸; Verma, Mitchell *et al.*, 2023 ▸; Guo *et al.*, 2023 ▸) as well as some model larger peptides such as insulin (Schlichtkrull *et al.*, 1957 ▸; Link & Heng, 2021 ▸; Link & Heng, 2022 ▸; Ferreira *et al.*, 2022 ▸) and vancomycin (Kim *et al.*, 2011 ▸; Li *et al.*, 2023 ▸). Additionally, crystallographic analysis has been performed on a variety of peptides for the exploration of subjects such as amyloid fibrils (Yoo *et al.*, 2016 ▸; Samdin *et al.*, 2023 ▸) and peptide antibiotics (Pfeffer *et al.*, 1991 ▸). For the family of GLP-1 and/or GIP analogue peptides, receptor-complexed crystal structures have been elucidated for GLP-1, GIP and exendin-4 (Underwood *et al.*, 2010 ▸; Parthier *et al.*, 2007 ▸; Runge *et al.*, 2008 ▸), as well as some analogues (including notable therapeutic peptides such as semaglutide; Lau *et al.*, 2015 ▸; Oddo *et al.*, 2018 ▸; Evers *et al.*, 2018 ▸). However, crystal structures for non-receptor-complexed peptides in this family are significantly more sparse. Crystallization has been demonstrated for GLP-1 (Kim & Haren, 1995 ▸) and the analogue glucagon-cex (Li *et al.*, 2007 ▸), and a crystal structure has been reported for a separate glucagon analogue (Sturm *et al.*, 1998 ▸). However, for conjugated GLP-1 peptides such as semaglutide and tirzepatide, which fall under the category of lipopeptides owing to the fatty-acid conjugation, there is a distinct lack of reported crystallization or crystal structures for the unbound (not complexed with the GLP-1 or GIP receptor) molecule (Knudsen & Lau, 2019 ▸).

In the present study, crystallization was initially explored in the context of assessing the feasibility of crystallization as a preparative strategy for a GLP-1/GIP lipopeptide, with single-crystal X-ray diffraction employed primarily to verify the nature of the crystalline phase and to gain insight into crystal packing and stability. However, the structure obtained from the diffraction experiments is believed to be the first reported crystal structure for an unbound (non-receptor-complexed) GLP-1/GIP analogue, motivating this work as a standalone crystallographic study.

## 2. Materials and methods

### 2.1. GG-353

**Figure 1:**
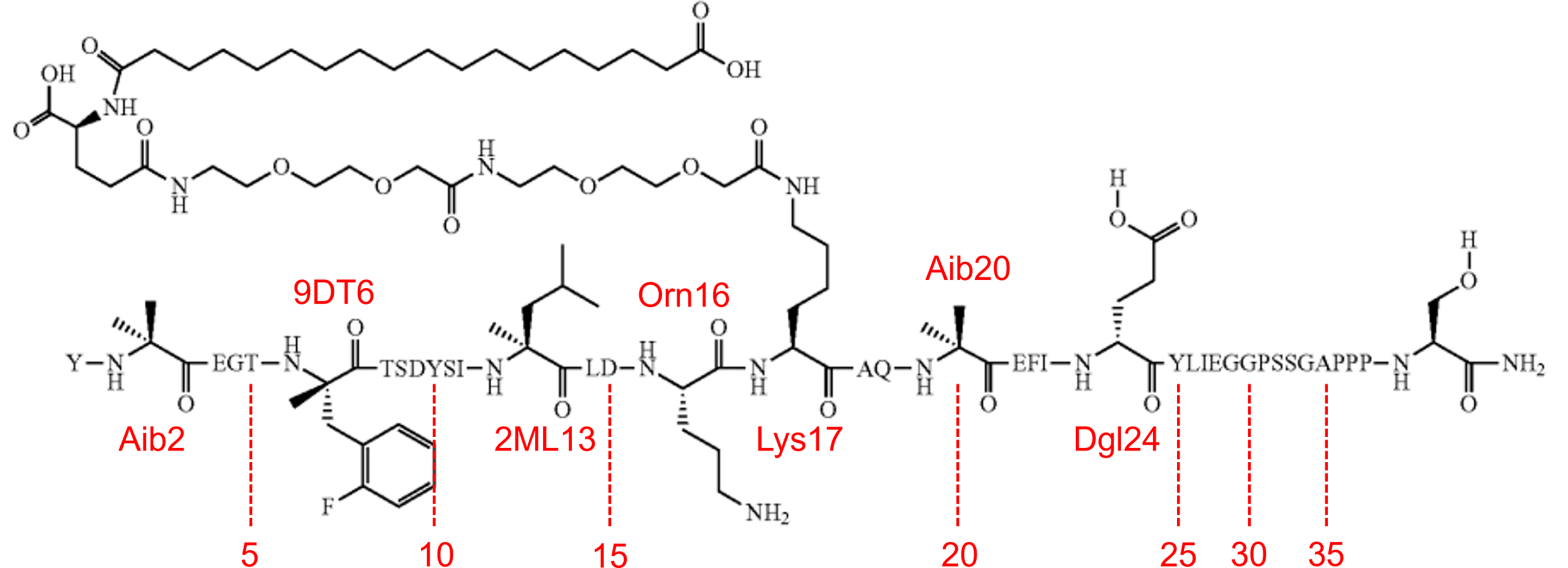
Molecular sequence of GG-353. Natural amino acids are represented by their single-letter codes; non-natural amino acids are shown in detail and labelled with their three-letter code and residue number.

GG-353 is a 42-amino-acid lipopeptide consisting of a 39-amino-acid backbone which incorporates sequence components based on the GLP-1 and GIP hormones. GG-353 is synthesized by solid-phase or solution-phase techniques; its sequence is presented in Fig. 1 ▸. The backbone contains multiple non-natural amino-acid residues, which are listed below including their corresponding three-letter codes.

*Position 2*: 2-aminoisobutyric acid, Aib.
*Position 6*: (2-fluoro)-α-methylphenylalanine, 9DT.
*Position 13*: α-methylleucine, 2ML.
*Position 16*: ornithine, Orn.
*Position 20*: 2-aminoisobutyric acid, Aib.
*Position 24*: d-glutamic acid, Dgl.

**Figure 2:**
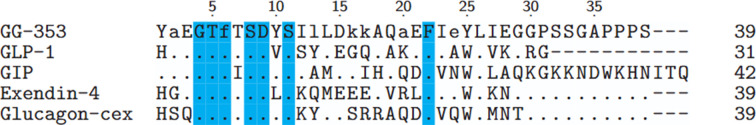
Aligned sequences for GG-353 compared with exendin-4, GLP-1 (7–37) and glucagon-cex. Non-natural amino acids have been replaced with their closest proteinogenic analogue (Ala2, Phe6, Leu13, Lys16, Lys17, Ala20 and Glu24) and are denoted in lower case. Matched residues to GG-353 are represented by a period, fully conserved residues are highlighted in blue and absent residues are represented by a dash.

Additionally, the Lys17 side chain is acylated to a conjugate molecule consisting of two 2-[2-(2-aminoethoxy)ethoxy]acetic acid (AEEA) linkers, a γ-glutamic acid spacer and a C18 fatty diacid (see Fig. 1 ▸). The conjugate molecule has a molecular weight of ∼730 g mol^−1^, while the molecular weight of GG-353 is ∼4920 g mol^−1^. The sequence of GG-353 is similar to that of both native GLP-1 (7–37) (Underwood *et al.*, 2010 ▸) and GIP (1–42) (Gallwitz, 2022 ▸), as well as glucagon-cex (Li *et al.*, 2007 ▸) and exendin-4 (Neidigh *et al.*, 2001 ▸); the sequences are compared in Fig. 2 ▸.

GG-353 was provided as a sodium salt by an industrial collaborator at a purity of ≥95% (with the identity and purity confirmed by the supplier via HPLC), and used as received without further purification. It was delivered as a white lyophilized powder and stored at −20°C prior to use. The purity and identity of the raw material were further verified in-house via LC/MS with a Shimadzu Nexera LCMS-2050, details of which can be found in the supporting information.

### 2.2. Crystallization

To make stock solutions, GG-353 powder was removed from the freezer and allowed to equilibrate at room temperature for at least 30 min before use, before being solubilized at concentrations of 70 and 100 mg ml^−1^ in purified deionized water provided by a PURELAB Chorus 1 (ELGA LabWater, High Wycombe, UK), followed by filtration through a 0.2 µm pore-size regenerated cellulose membrane filter (Sartorius Minisart RC4) to remove any solid contaminants. GG-353 was found to be readily soluble in water (≥200 mg GG-353 per millitre of water), so no pH adjustment or buffer preparation was required at this stage. The pH as prepared was determined using a Mettler Toledo LE420 electrode to be 7.4 at room temperature. The solutions were stored at 4°C when not in use, and allowed to warm to room temperature for ≥30 min before use in screens.

#### 2.2.1. Initial screening

Initial crystallization attempts were performed using the commercially available IndexHT (referred to here as ‘Index’) and PEG/IonHT (‘PEGIon’) kits from Hampton Research, as well as the MemGold2 HT-96 (‘MemGold2’) kit from Molecular Dimensions. Crystallization experiments were performed in a 96-well sitting-drop configuration using SWISSCI MRC 2 Lens crystallization plates. Each reservoir was initially filled with 100 µl reservoir solution from the kits. Sitting drops were dispensed using an SPT Labtech Mosquito LCP (Melbourn, UK), with each drop containing 100 nl GG-353 stock solution and 100 nl reservoir (precipitant) solution. The plates were sealed and incubated at 298 K.

#### 2.2.2. Microseed matrix screening

Crystals generated from subsequent unseeded sitting-drop vapour-diffusion optimization experiments were used to create a seed stock of GG-353 crystals. Optimization of the initial hit condition from screening proved to be a difficult task owing to issues with poor reproducibility. A detailed description of attempts to optimize these conditions is given elsewhere (Mitchell *et al.*, 2026 ▸). Briefly, the crystals used for the seed stock were grown via sitting-drop vapour diffusion at the 4 µl scale, which comprised 2 µl 70 mg ml^−1^ GG-353 stock solution in water and 2 µl precipitant/reservoir solution containing 0.1 *M* bis-Tris pH 5.3, 0.2 *M* ammonium acetate, 25%(*w*/*v*) PEG 3350. The methodology for creating the seed stock was similar to that outlined elsewhere (D’Arcy *et al.*, 2014 ▸) and is given in detail in the supporting information.

To incorporate the seed stock into crystallization screens, an identical procedure was followed to that of the initial screening, but with the drops consisting of 100 nl 70 mg ml^−1^ GG-353 stock solution, 80 nl reservoir solution and 20 nl seed stock. Unseeded experiments (100 + 100 nl) were also carried out in parallel to be able to compare seeded and unseeded experiments directly. The plates were again sealed and incubated at 298 K; this temperature was chosen as optimization experiments of the unseeded crystallization conditions (which are covered in detail elsewhere; Mitchell *et al.*, 2026 ▸) showed a preference for this temperature over the more conventionally used 293 K.

### 2.3. Data collection and analysis

Prior to crystallographic data collection, single crystals were isolated from the drop and flash-cooled in liquid nitrogen. For crystals obtained in precipitant solutions which did not contain a suitable cryoprotectant, the crystals were first soaked in a 70:30 mixture of precipitant solution:ethylene glycol before being cooled.

Single-crystal X-ray diffraction studies were performed on the I24 tuneable microfocus beamline at Diamond Light Source (Oxford, UK) using a CdTe EIGER2 9M detector set to a wavelength of 0.62 Å. 360° of data were collected for each crystal, with an oscillation of 0.1° and exposure of 0.05 s per diffraction image. The transmission was fixed at 20%, with a beam size of 7 × 7 µm and a crystal-to-detector distance of 193.9 mm. Data were collected at 100 K.

Diffraction data were processed using the built-in data-processing pipeline at Diamond Light Source. The data were indexed and reduced using *fast_dp* (Winter & McAuley, 2011 ▸), *xia*2 (Winter, 2010 ▸) and *autoPROC* (Vonrhein *et al.*, 2011 ▸). If the indexed, reduced data were of sufficient quality, they were automatically sent for model building and refinement via molecular replacement using the *MrBUMP* pipeline (Keegan & Winn, 2008 ▸). Diffraction data and the resulting density maps and structures were also processed manually using the *CCP*4*i*2 (Potterton *et al.*, 2018 ▸) and *CCP*4 Cloud (Krissinel *et al.*, 2022 ▸) software; data reduction was performed using *DIALS* (Winter *et al.*, 2018 ▸) with merging and analysis from *AIMLESS* (Evans & Murshudov, 2013 ▸), and molecular replacement was carried out with *Phaser* (McCoy *et al.*, 2007 ▸) with the initial model generated by *MrBUMP*. Model building was performed using *Buccaneer* (Cowtan, 2006 ▸) and *ModelCraft* (Bond & Cowtan, 2022 ▸), model refinement was performed using *REFMAC* (Murshudov *et al.*, 1997 ▸) and manual model building, particularly, the incorporation of non-natural amino acids into the peptide sequence, was performed using *Coot* (Emsley & Cowtan, 2004 ▸). Model visualization and validation were carried out using *CCP*4*MG* (McNicholas *et al.*, 2011 ▸) and *Iris* (Rochira & Agirre, 2021 ▸), respectively.

The incorporation of non-natural amino acids was for the most part a simple process, as α-methylalanine, ornithine, α-methylleucine, (2-fluoro)-α-methylphenylalanine and d-glutamic acid were included in the monomer libraries available in *Coot* and thus could be added via mutation of the naturalized sequence. Addition of the fatty acid conjugated to the side-chain amine on Lys17 was performed by defining the entire conjugate molecule (AEEA-AEEA-γGlu-C18 diacid) as a ligand using *AceDRG* and connecting the ligand to Lys17 with a defined amide bond via *AceDRG*.

## 3. Results and discussion

### 3.1. Initial screening

**Figure 3:**
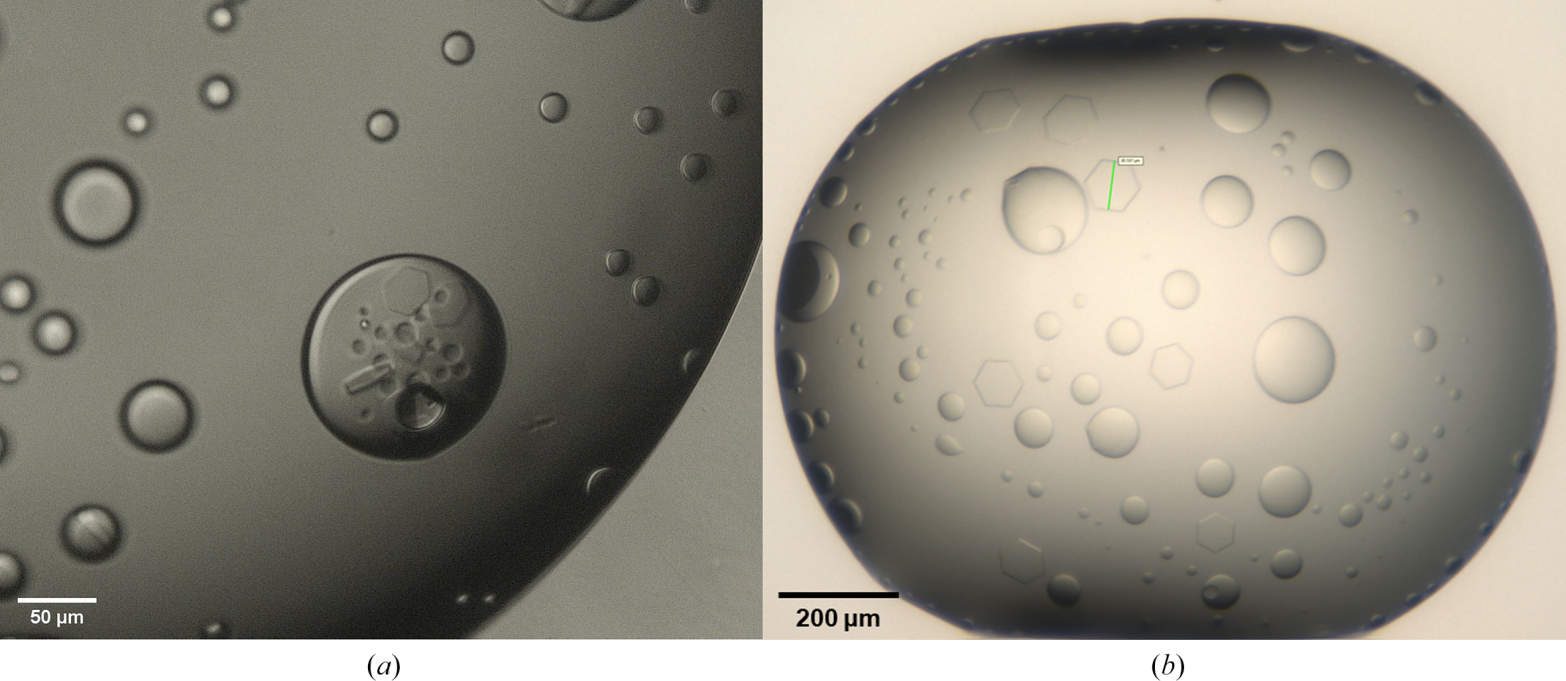
(*a*) Hexagonal and rod-like crystals of GG-353 observed during initial crystallization screening. (*b*) Hexagonal crystals of GG-353 grown in unseeded optimization experiments. The green line (short diagonal) represents approximately 80 µm.

After 17 days, crystals were observed (see Fig. 3 ▸*a*) in condition G6 of the Index screen [0.1 *M* bis-Tris pH 5.5, 0.2 *M* ammonium acetate, 25%(*w*/*v*) PEG 3350] at GG-353 stock concentrations of both 70 and 100 mg ml^−1^. Crystals grew as hexagonal and rod-shaped plates inside a pre-existing coacervate phase, which was determined via UV microscopy to be comparatively richer in GG-353 than the bulk phase.

The conditions discovered from initial screening were subsequently optimized (details of this optimization can be found elsewhere; Mitchell *et al.*, 2026 ▸), with regular hexagonal plate crystals observed to grow up to 80 µm along their short diagonal (Fig. 3 ▸*b*). However, little-to-no diffraction was observed during single-crystal X-ray studies. It was also observed that the crystals were exceptionally fragile owing to their habit, and behaved in a gel-like manner, indicating a high solvent content, which may be the reason for the lack of diffraction (McPherson & Cudney, 2014 ▸).

### 3.2. Microseed matrix screening

**Figure 4:**
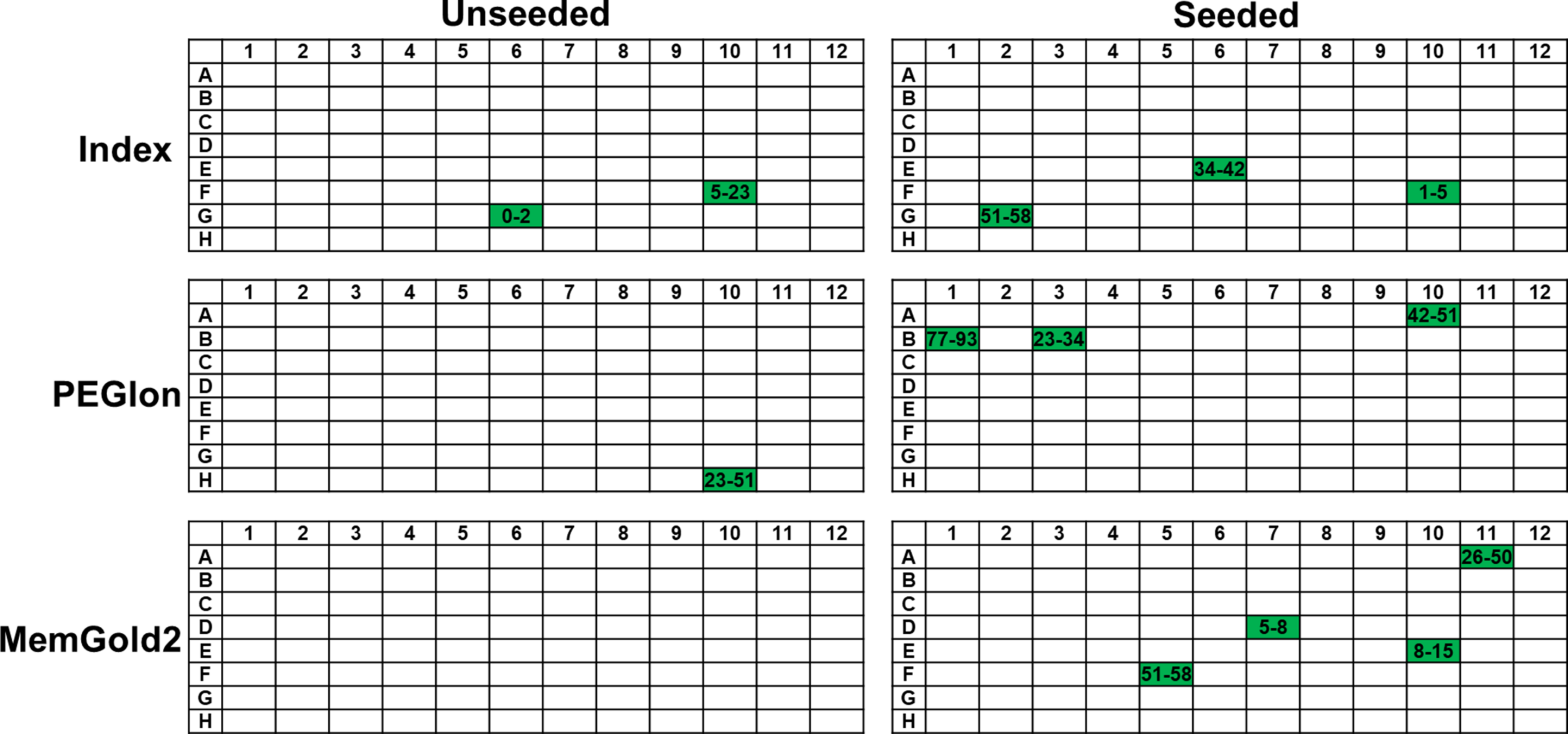
Comparison of unseeded and seeded screening results using Index, PEGIon and MemGold2 sparse-matrix screens. Crystalline hits are highlighted in green, and the time taken to observe crystals (in days) is given within the highlighted cell. The reservoir/precipitant conditions for each hit are given in Table 1 ▸.

Microseed matrix screening increased the number of hits observed during sparse-matrix screening significantly over the duration of the seeded experiments. As can be seen in Fig. 4 ▸, while a further two hits were discovered in the unseeded screens (for a total of three over the 288 conditions studied), seeding resulted in ten unique conditions which were conducive to crystal growth. Images of all crystals observed during microseed matrix-screening experiments, including those produced without seeding, are provided in the supporting information. The reservoir/precipitant conditions for each hit are given in Table 1 ▸.

These conditions can be broadly divided into two categories: (i) conditions which resulted in the growth of hexagonal plate-like crystals as previously observed and (ii) conditions which resulted in the observation of new crystal habits. In conjunction with seeding, crystals were obtained in a variety of buffers and buffer pHs (4.5–7), salts and PEG molecular weights (400–6000); a vast increase when compared with the hits obtained from unseeded experiments, which were limited to a single buffer (bis-Tris pH 5.5) and molecular-weight PEG (3350). Similarly to protein crystallization (D’Arcy *et al.*, 2014 ▸), the use of microseed matrix screening is a material-sparse and efficient method for uncovering and optimizing crystallization conditions for peptides.

**Figure 5:**
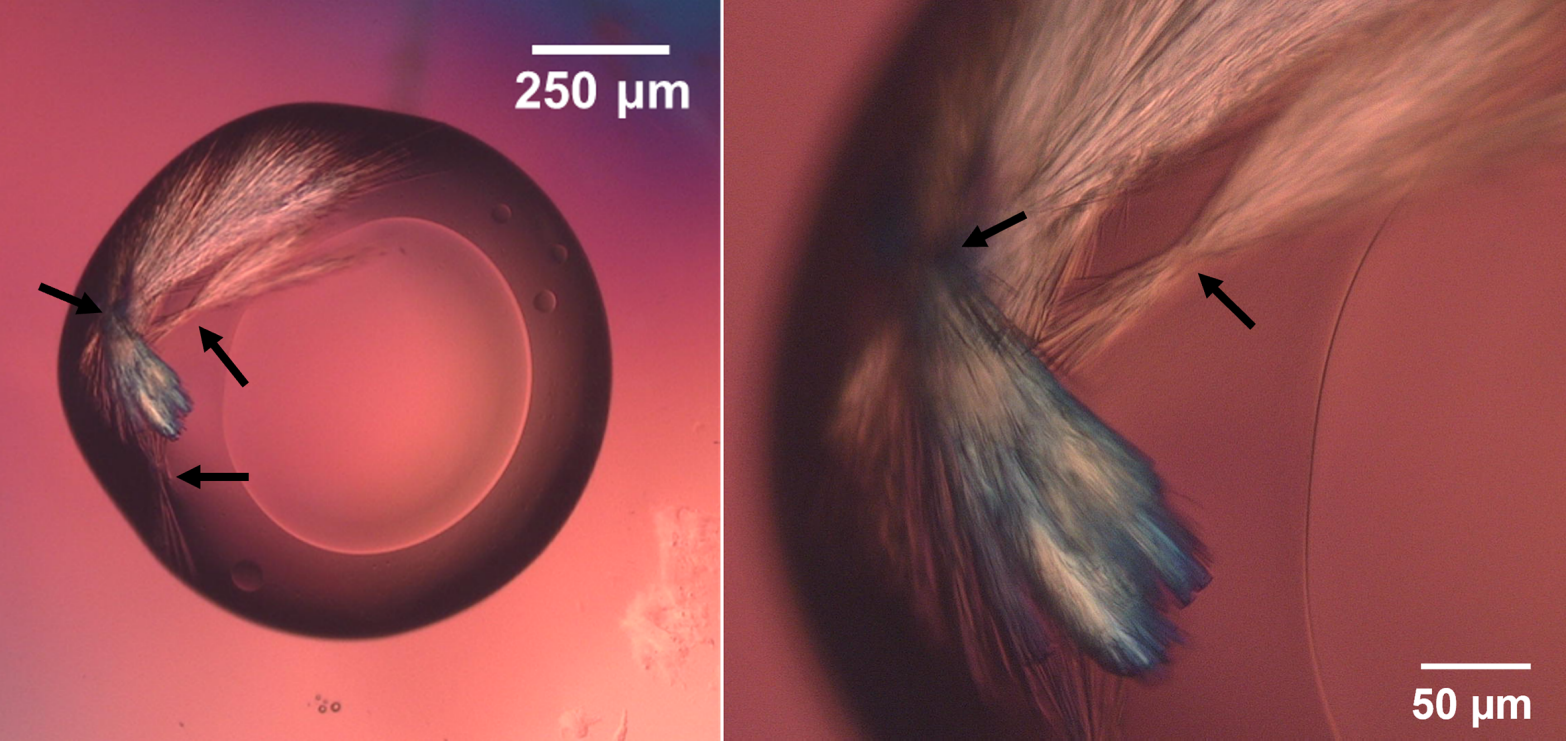
Clusters of fine acicular crystals exhibiting strong birefringence under polarized light [reservoir condition: 0.1 *M* sodium citrate pH 4.5, 0.2 *M* ammonium phosphate monobasic, 0.1 *M* ammonium sulfate, 32%(*v*/*v*) PEG 400]. Postulated positions of seed material are indicated with arrows.

Of particular interest were the new crystal habits uncovered during MMS. While hexagonal crystals of a suitable size for single-crystal analysis were obtained from seeded conditions (as well as two new unseeded conditions), subsequent diffraction experiments resulted in a lack of improvement, with little-to-no diffraction observed. However, the crystals obtained in condition A11 of MemGold2, while initially appearing as a poor candidate for optimization, owing to the dense clusters of acicular crystals (Fig. 5 ▸), exhibited a dramatic improvement in diffraction quality. It was also noted while harvesting these crystals for analysis that they exhibited improved mechanical integrity, signifying that the solvent content was significantly reduced and evidencing a change in polymorph via MMS. The improved mechanical integrity also suggested that these crystals produced by seeding may be a more viable candidate for further optimization, especially with the view of attempting to scale towards batch crystallization for purification purposes. It is unknown whether the seed material acts as a ‘classical’ seed which grows upon addition to a supersaturated solution, or via epitaxial jumps (Stura *et al.*, 1999 ▸) whereby a new polymorph nucleates and grows on the surface of the seed crystal due to favourable epitaxy between the two polymorphs. Unlike the crystals from which the seed stock was produced, these acicular crystals also exhibited strong birefringence under cross-polarized light (see Fig. 5 ▸). Unfortunately, the lack of diffraction of the initial crystals prevented a true understanding of potential polymorphism.

### 3.3. Single-crystal X-ray data collection

Where of suitable size, crystals were harvested from both unseeded and seeded conditions for single-crystal diffraction experiments. For the plate-like crystals, the degree of diffraction was either very poor or absent, indicating that the diffraction quality of the plate-like crystals had not notably improved through MMS. The crystals from PEGIon condition B3 showed the best improvement, with diffraction to ∼10 Å resolution but low overall completeness (∼30%). Initial indexing of the diffraction data gave *a* = *b* = 168.21, *c* = 180.33 Å, α = β = 90, γ = 120°. The volume of the unit cell was excessively large compared with the molecular weight of GG-353; Matthews coefficient analysis suggested that there were an enormous number (∼250–500) of monomers in the asymmetric unit. Ultimately, the poor quality of the diffraction data obtained prevented any meaningful interpretation.

However, a crystal from MemGold2 condition A11 diffracted to a relatively high resolution (∼1.6 Å as determined by a fitted CC_1/2_ > 0.3 cutoff in *DIALS*). A single 360° sweep was found to provide a full (≥99.8% complete) dataset, with the automatic data-reduction protocols suggesting space group *P*4_1_, resulting in ambiguity between the *P*4_1_/*P*4_3_ enantiomorphic pair. Subsequent initial map inspection revealed that the true space group was *P*4_3_, which was then fixed for subsequent manual reprocessing of the diffraction data. Calculation of the Matthews coefficient resulted in an estimated solvent content of ∼49%, corresponding to one monomer in the asymmetric unit. The data-reduction statistics for the dataset collected are given in Table 2 ▸; per-shell statistics can be found in the supporting information.

### 3.4. Structure solution

**Figure 6:**
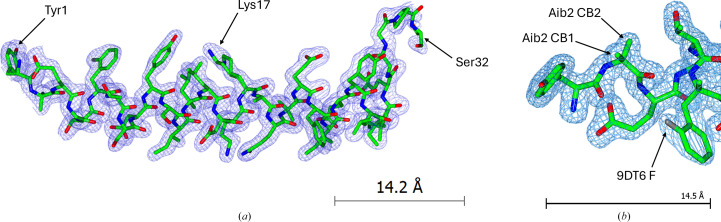
(*a*) Final molecular-replacement model fit to the experimentally obtained electron-density map via diffraction data. (*b*) Resolution of additional atoms in the nonstandard amino acids Aib2 and 9DT6.

Automated model building initially gave a poor solution to the experimentally determined electron-density map, due to the aforementioned enantiomorphic ambiguity of the space group. With the data re-indexed in the true space group (*P*4_3_), it was then possible to obtain a much better model fit. Among the tested search models in *MrBUMP*, the template derived from PDB entry 2qkh (60% sequence identity) gave the best solution, with a TFZ score of 4.5 and a LLG of 28.0, and was subsequently refined. The maps were of sufficient quality to manually build all non-natural amino acids (positions 2, 6, 13, 16, 20 and 24); many of these modifications were visible directly from the difference density maps. However, attempts to build the model out past Ser32 were largely unsuccessful owing to a lack of continuous electron density (see Fig. 6 ▸). Similarly, attempts to model the conjugate molecule on Lys17 were also not possible. This was reasoned to be due to high conformational flexibility for both the amidated C-terminus (Ser33–Ser39) and the conjugate molecule. This result is in line with those obtained from cryo-EM studies for tirzepatide (which contains an identical amidated C-terminal composition and is similarly acylated) in complex with both the GLP-1 and GIP receptors (Zhao *et al.*, 2022 ▸), which similarly were not able to resolve the lipid chain, as well as solution NMR studies of exendin-4 and GLP-1 analogues (Evers *et al.*, 2017 ▸, 2018 ▸), which both show significant flexibility as per the ensemble. For the amidated C-terminal residues (Ser33–Ser39), the theory of high conformational flexibility corroborates with a notable increase in the isotropic *B* factor for Pro31 (39.2 Å^2^) and Ser32 (71.2 Å^2^) compared with the average *B* factor for the rest of the chain (21.8 Å^2^). For the conjugate molecule, patches of difference density were observed in the vicinity of Lys17, but a lack of clear connection and the observed conformational flexibility of the side chain on Lys17 (as shown by partial occupancy of the side chain and the two resultant alternate conformations (see Fig. 6 ▸) prevented meaningful interpretation of these difference maps. Additionally, the components present in the crystallization cocktail (namely PEG 400 and PEG 3350) would present similar conformational flexibility and could also be part of the crystal structure, so the resultant ambiguity further prevented building of the conjugate molecule. Ultimately, it was not possible to prove the existence of the lipid chain or the amidated C-terminal residues Ser33–Ser39, and therefore the intactness of the peptide, solely via crystallographic data.

**Figure 7:**
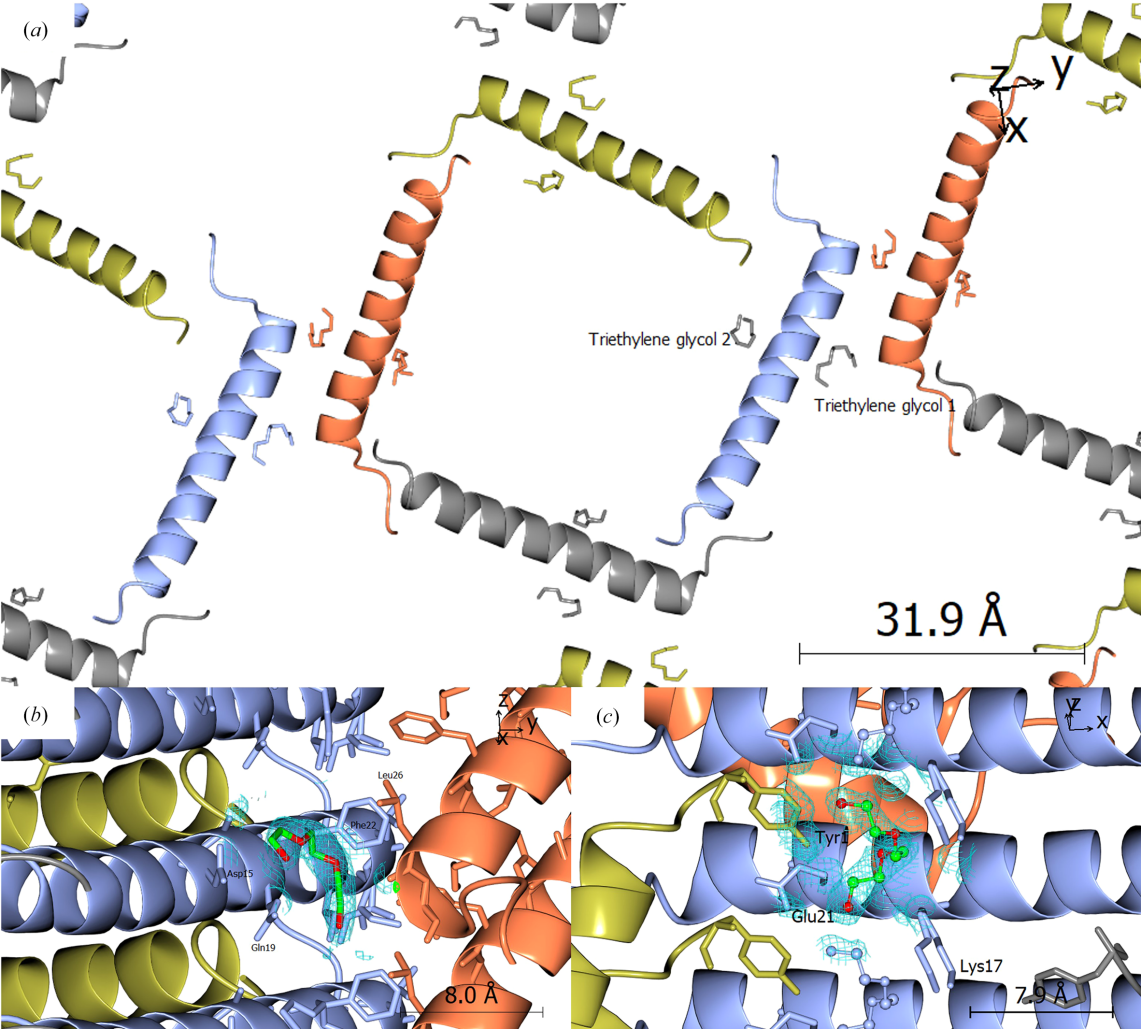
(*a*) Positions of triethylene glycol ‘ligands’ fitted to the density map and their corresponding positions in the crystal lattice. (*b*) Position of triethylene glycol 1 in the density map. (*c*) Position of triethylene glycol 2 in the density map.

As a result, the conjugate molecule was removed from model building, and where sufficiently long continuous sections of density were observed, triethylene glycol molecules were added as ligands to represent either the conjugate molecule or any PEG molecules which may be part of the crystal structure. A total of two triethylene glycol molecules were added as ligands to represent density in the ‘corners’ of the crystal structure (see Fig. 7 ▸): one in the vicinity of Lys17 and another on the opposite side of the peptide chain. Due to the geometry of the crystal structure, the second ligand is unlikely to be part of the conjugate molecule, and is postulated to instead be a fragment of a PEG molecule present in the crystallization liquor (either PEG 400 or PEG 3350). Further refinement led to final *R* and *R*_free_ values of 0.208 and 0.245, respectively. The full list of refinement statistics are provided in Table 3 ▸.

It is worth noting that the refinement statistics for the GG-353 structure appear to be slightly anomalous at first glance, notably the higher-than-expected *R*_free_ value given the resolution of the data, and the relatively high percentage of Ramachandran outliers. The heightened *R*_free_ value can be attributed to the aforementioned positive density, especially in the vicinity of Lys17, which could not be explained by the model. As a result, it was deemed more appropriate to leave these patches of density unmodelled rather than attempting to spuriously fit more ligands to ‘improve’ the model. Additionally, inspection of the Ramachandran outliers using *Iris* showed that the only Ramachandran outlier was Gly30. Manual inspection of the model and map revealed that the density was clear in the vicinity of Gly30 and no other conformation was possible. To attempt to improve the model statistics, paired refinement via *PAIREF* (Malý *et al.*, 2020 ▸) was attempted at resolutions of 2.5–1.5 Å, as *AIMLESS* had estimated resolution limits of 2.00 Å [based on overall 〈*I*/σ(*I*)〉 > 2] to 1.59 Å [based on 〈*I*/σ(*I*)〉 > 1 along the *l* axis]. The resultant statistics (namely *R* and *R*_free_) appeared ‘better’ at the suggested resolution cutoff of 2.5 Å, owing to the reduced noise in the maps and the higher values of *R* and *R*_free_ associated with lower resolution data, but this came at the penalty of a less detailed map; for example, the alternative conformations of Ile27 were no longer visible. As such, paired refinement was not pursued further and the resolution of the data was kept at 1.59 Å. It can be argued that the value of CC_1/2_ in the highest resolution shell at this resolution (see Table 2 ▸) is low, making the resolution choice difficult to justify. However, literature dedicated to resolution cutoffs in crystallographic analysis indicate that a statistic which can be reliably used to indicate resolution cutoff does not exist (Karplus & Diederichs, 2012 ▸; Dubach & Guskov, 2020 ▸) and that ‘optimal’ values for CC_1/2_ may lie between 0.1 and 0.5 (Dubach & Guskov, 2020 ▸).

**Figure 8:**
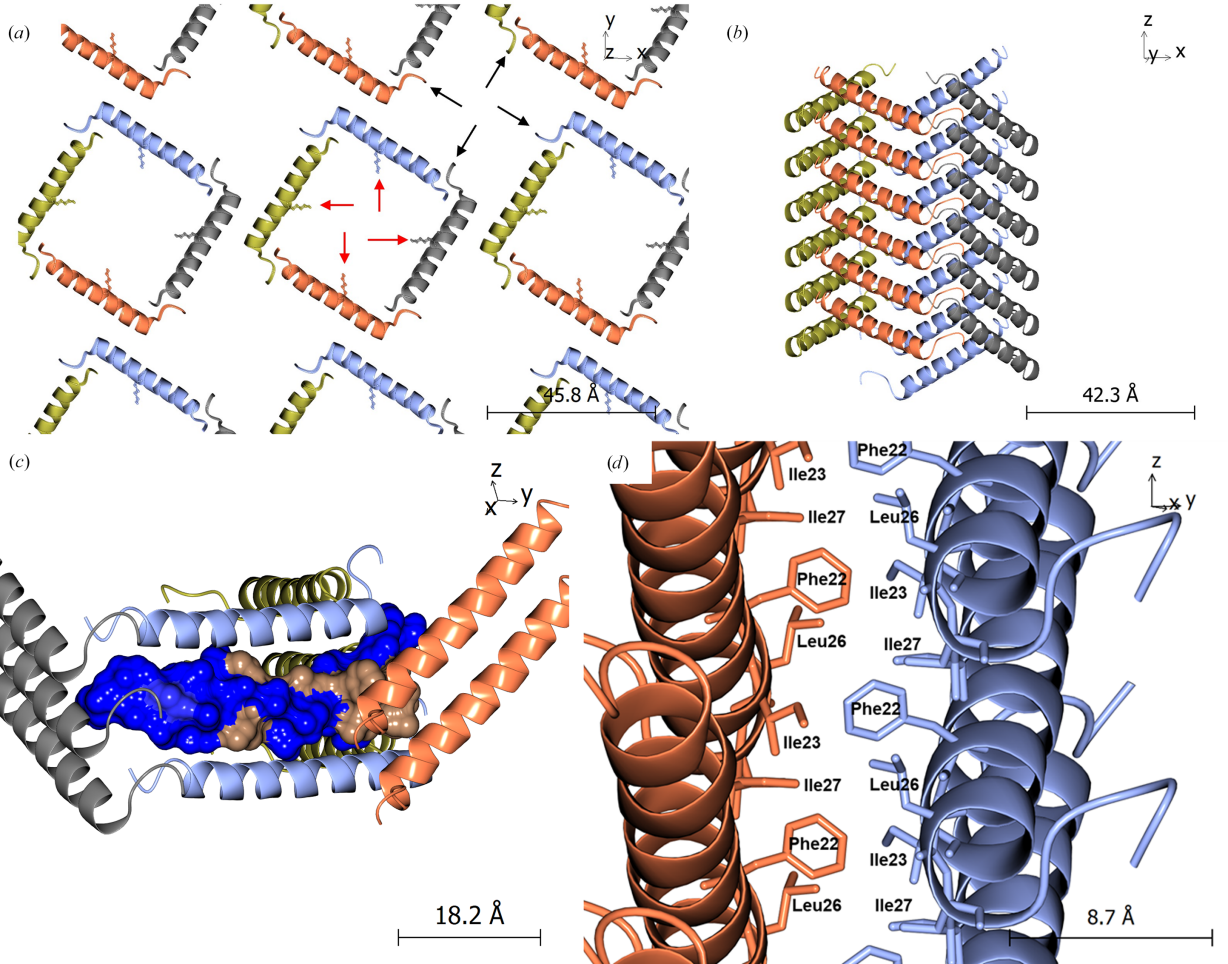
Experimentally determined crystal structure of GG-353. (*a*) Square channels formed down the *c* axis of the crystal structure, with channel formation facilitated by hydrogen-bonding interactions between terminal residues. Positions of attachment of the flexible fatty-acid conjugate molecule onto Lys17 are indicated with red arrows; positions of attachment of flexible amidated C-terminal residues are indicated with black arrows. (*b*) Pore formation is facilitated by stacking of the helical peptide portions to form clockwise-ascending spirals. GG-353 monomers are coloured by crystal symmetry. (*c*) Hydrophobicity of a GG-353 molecule superimposed into the crystal packing, with hydrophobic regions (beige) buried within the crystal structure and hydrophilic regions (blue) exposed to solvent channels. (*d*) C⋯H—π interactions around Phe22 (with Ile23, Leu26 and Ile27) forming the primary crystal contact between square motifs in the lattice.

As expected from analysis of other GLP-1 analogue peptides, GG-353 contains an α-helical motif running from Gly4 to Glu28, with the remaining sections being randomly coiled. However, the crystal structure of GG-353 is quite interesting; monomers of GG-353 form square motifs within the crystal lattice, with the N-terminus of one monomer lying next to the amidated C-terminus of the next (see Fig. 8 ▸). These square motifs are helical in and of themselves, forming a clockwise-ascending spiral structure, with the helical core of each monomer stacking to form square channels running through the crystal lattice. As a result, the crystal lattice is inherently porous, with the square pores being approximately 30 × 30 Å in area and extending infinitely down the *c* axis of the crystal. This type of crystal packing is reminiscent of that of a macrocyclic β-sheet peptide (Yoo *et al.*, 2016 ▸). It is worth noting, however, that these porous regions would also contain the disordered sections of the peptide, namely the amidated C-terminal residues and the conjugate molecule. As such, the accessible volume of the pore would be significantly lower than the total volume available, and any species which diffuse into the pores in the crystal lattice (*e.g.* water, salts) would also interact with the disordered, flexible regions of the peptide within the lattice. Due to the crystallographic symmetry, the pores are not identical; each pore either contains conjugate molecules or amidated C-terminal residues (Ser33–Ser39), but not both (see Fig. 8 ▸). As the conjugate molecule is primarily composed of the aliphatic chain of the fatty acid, it is expected that the hydrophobicity of the pores containing the amidated C-terminal residues would be lower than that of the pores containing the fatty-acid conjugate molecule. It is also worth noting that while the conjugate molecule and C-terminal residues were omitted from the final model, it was possible to fit them into the crystal structure without atomic clashes, reinforcing the idea that flexible sections of the peptide are still present in the crystal structure.

As shown in Fig. 8 ▸(*d*), the primary crystal contact between neighbouring square motifs exists in the form of C⋯H—π interactions around Phe22 between neighbouring chains, which simultaneously act to bury the hydrophobic section of GG-353 within the crystal lattice and reduce the exposure of hydrophobic groups to solvent. In this region, Phe22 interacts with other hydrophobic residues, namely Ile23, Leu26 and Ile27, both within and between chains. The occurrence of this crystal contact is in line with results postulated by Kim and Haren, who observed that the addition of aromatic compounds (*e.g.* phenol) to crystals of insulinotropin [GLP-1(7–37)] resulted in the transition to an amorphous state, and postulated that this was due to the disturbance of lattice interactions in this hydrophobic region around Phe22 (Kim & Haren, 1995), which this crystal structure supports. These hydrophobic interactions about Phe residues have also been observed in the 3 Å resolution crystal structure of another glucagon analogue (Sturm *et al.*, 1998 ▸). In contrast, the stacking of helices along the *c* axis is facilitated by salt bridges, such as those between the side-chain amine of Orn16 with the side-chain carboxyl group of Asp15, as well as the N-terminus with the side-chain carboxyl group of Glu21. This is bolstered by hydrogen-bonding interactions along the chain, such as those between the main-chain amide NH of Phe22 with the side-chain carboxyl group of d-Glu24, the side-chain amine of Orn16 with the side-chain hydroxyl group of Ser11, and the main-chain carboxyl of Gly30 with the main-chain amide NHs of Glu3 and Gly4.

This crystal structure is therefore unique; unlike proteins, which are large enough to be able to sequester flexible or hydrophobic regions within their tertiary structure, smaller peptides such as GG-353 are inherently solvent-exposed throughout the chain. As such, the crystal lattice itself accounts for these otherwise deleterious features by burying them within the crystal contacts (as shown by Phe22) or by allowing them to occupy void spaces within the lattice (as in the case of the amidated C-terminal Ser33–Ser39 residues and fatty-acid conjugate molecule on Lys17). The difficulty in obtaining a crystal structure for GG-353 may therefore lie in its low crystallization propensity, as a result of its inherent disorder in solution. This theory corroborates with previous experimental crystallization screening of glucagon-cex, which does not contain a fatty acid and was found to crystallize readily from solution (Li *et al.*, 2007 ▸), as well as a similar glucagon analogue which was similarly not acylated (Sturm *et al.*, 1998 ▸).

## 4. Conclusions

Herein, the crystal structure of a noncomplexed GLP-1/GIP analogue lipopeptide is reported for the first time. The use of a single round of microseed matrix screening was found to be a powerful tool to overcome diffraction issues, which conventional unseeded optimization approaches could not. It is recommended that similarly to proteins, microseed matrix screening should be attempted as soon as feasibly possible for peptides, as it is a simple but powerful optimization method. The use of microseed matrix screening was also found to improve important crystal-quality attributes for the goal of purification, signifying its potential utility both for crystallo­graphic and bulk-scale crystallization purposes.

In terms of crystal structure, the peptide forms a unique crystal lattice composed of square pores which facilitate flexible sections of the peptide to remain disordered. Analysis of the crystal-packing interactions reveals that while the stacking of helical sections of neighbouring peptides is mediated by hydrogen bonding, the primary crystal contact between the square pores is aromatic C⋯H—π interactions of Phe22. This crystal structure serves as a proof-of-concept for the feasibility of crystallization of acylated or otherwise conjugated GLP-1 analogue peptides as well as providing insight into critical residues for the formation of important crystal contacts. Therefore, the results reported herein warrant further investigation into developing crystallization as a purification process for GLP-1 analogue (lipo)peptides, particularly related to transitioning from the nanolitre-scale vapour-diffusion experiments performed to batch or continuous crystallization setups on millilitre or litre scales. The dramatic improvement in diffraction quality via microseed matrix screening also suggests that further optimization of the new seeded condition would be fruitful in further improving crystal quality.

## Supplementary Material

PDB reference: acylated GLP-1/GIP analogue peptide, 9tb1

LS/MS analysis, seed preparation protocol, crystal images, per-shell data reduction statistics and Ramachandran analysis, including Supplementary Tables and Figures. DOI: 10.1107/S2053230X26001937/va5069sup1.pdf

## Figures and Tables

**Table 1:** Reservoir conditions which produced hits during microseed matrix screening Conditions which produced crystals identical to previous optimization attempts are in bold.

Screen/well	Reservoir conditions
Unseeded	
**Index F10**	**0.1 *M* bis-Tris pH 5.5, 0.2 *M* NaCl, 25%(*w*/*v*) PEG 3350**
**Index G6**	**0.1 *M* bis-Tris pH 5.5, 0.2 *M* ammonium acetate, 25%(*w*/*v*) PEG 3350**
**PEGIon H10**	**0.2 *M* NaBr, 25%(*w*/*v*) PEG 3350**
Seeded	
Index E6	0.1 *M* bis-Tris pH 6.5, 0.05 *M* CaCl_2_·2H_2_O, 30%(*v*/*v*) PEG MME 550
Index F10	0.1 *M* bis-Tris pH 5.5, 0.2 *M* NaCl, 25%(*w*/*v*) PEG 3350
**Index G2**	**0.1 *M* bis-Tris pH 5.5, 0.2 *M* LiNO_3_·H_2_O, 25%(*w*/*v*) PEG 3350**
**PEGIon A10**	**0.2 *M* NaI, 20%(*w*/*v*) PEG 3350**
**PEGIon B1**	**0.2 *M* NaSCN, 20%(*w*/*v*) PEG 3350**
**PEGIon B3**	**0.2 *M* LiNO_3_, 20%(*w*/*v*) PEG 3350**
MemGold2 A11	0.1 *M* sodium citrate pH 4.5, 0.2 *M* ammonium phosphate monobasic, 0.1 *M* ammonium sulfate, 32%(*v*/*v*) PEG 400
**MemGold2 D7**	**0.05 *M* MOPS pH 7, 0.05 *M* NaCl, 19%(*w*/*v*) PEG 6000**
MemGold2 E10	0.05 *M* sodium acetate pH 4.5, 0.23 *M* NaCl, 33%(*v*/*v*) PEG 400
**MemGold2 F5**	**0.1 *M* sodium citrate pH 5.5, 0.34 *M* ammonium sulfate, 12%(*w*/*v*) PEG 4000**

**Table 2:** Crystallographic statistics for GG-353 crystals after data reduction, as reported from *AIMLESS* (mosaicity parameters taken from *DIALS*)

Shell	Overall	Low	High
Space group	*P*4_3_		
*a*, *b*, *c* (Å)	64.66, 64.66, 11.42		
α, β, γ (°)	90, 90, 90		
Mosaicity (°)	σ_b_ = 0.031, σ_m_ = 0.353		
Resolution (Å)	64.66–1.59	64.66–8.71	1.62–1.59
Completeness (%)	99.8	99.4	100.0
CC_1/2_	0.988	0.997	0.173
*R*_meas_	0.312	0.093	3.448
*R*_p.i.m._	0.124	0.045	1.391
No. of observations	43020	249	2214
No. of unique reflections	6855	62	353
Multiplicity	6.3	4.0	6.3
Mean *I*/σ(*I*)	3.8	9.8	0.7

**Table 3:** Refinement statistics for the final GG-353 model

Resolution (Å)	1.59
No. of reflections	6852
No. of free reflections	674
*R*/*R*_free_	0.208/0.245
No. of (non-H) atoms	
Peptide	266
Water	15
Ligand	20
Mean *B* factors (Å^2^)	
Peptide	22.8
Water	49.2
Ligand	46.0
R.m.s. deviations	
Bond lengths (Å)	0.0107
Bond angles (°)	2.05
Ramachandran favoured residues (%)	92.31
Ramachandran outlier residues (%)	7.69

## Data Availability

The associated structure for GG-353 has been deposited within the PDB (PDB entry 9tb1).
