# Supplementary material for: Crystallization and 1.6 Å resolution crystal structure of an acylated GLP-1/GIP analogue peptide
Source: Acta Crystallogr F Struct Biol Commun. 2026 Mar 17;82(Pt 4):114–24. doi: 10.1107/S2053230X26001937 (PMC13041627; doi:10.1107/S2053230X26001937)
Supplement: Supplementary file 1 [file f-82-00114-sup1.pdf]

STRUCTURAL BIOLOGY COMMUNICATIONS

Volume 82 (2026)

Supporting information for article:

Crystallization and 1.6 Å resolution crystal structure of an acylated GLP-1/GIP analogue peptide

Hamish M. Mitchell, Boguslaw Nocek, Emily J. Guinn and Jerry Y. Y. Heng

S1. LC/MS Analysis

LC/MS was performed using a Shimadzu LC-40 and LCMS-2050 system. The LC-40 consisted of a DGU-405 degassing unit, an LC-40D XS solvent delivery system, an SIL-40C XS autosampler, a CBM-40 system controller, a CTO-40C column oven, and an SPD-M40 photo diode array detector. Information on the method used is given in Table S1.

Table S1: Parameters for the LC/MS method used to analyse the GG-353 raw material.

Liquid chromatography parameters			
Column	Phenomenex Luna® 5 µm C18(2), 100Å pore size, 250 x 4.6 mm		
Column temperature	16 °C		
Sample concentration	0.1 mg/mL in water		
Injection volume	25 µL		
Sample temperature	16 °C		
Flowrate	1 mL/min		
Mobile phase A	Water with 0.2% formic acid		
Mobile phase B	Acetonitrile with 0.2% formic acid		
Method			
Time	%A	%B	Curve
Initial	95	5	0

2.5	95	5	0
22.5	5	95	0
23	5	95	0
MS parameters			
Desolvation temperature	450 °C		
Nebulising gas flowrate	1.5 L/min		
Drying gas flowrate	4 L/min		
Heating gas flowrate	6 L/min		
Acquisition rate	0.5 Hz		
Mass range	200 m/z - 2000 m/z		

The total ion chromatograph is presented in Figure S1, and mass spectra of the major peaks observed at t = 2.329 mins, t = 14.578 mins, t = 14.959 mins, and t = 15.576 mins are presented in Figures S2, S3, S4, and S5, respectively. Additionally, extracted ion chromatographs (XICs) for the intact peptide (denoted as M) and the deconjugated peptide (denoted as M') are presented in Figures S6 and S7, and the 214 nm and 280 nm UV-Vis chromatograms are presented in Figure S8.

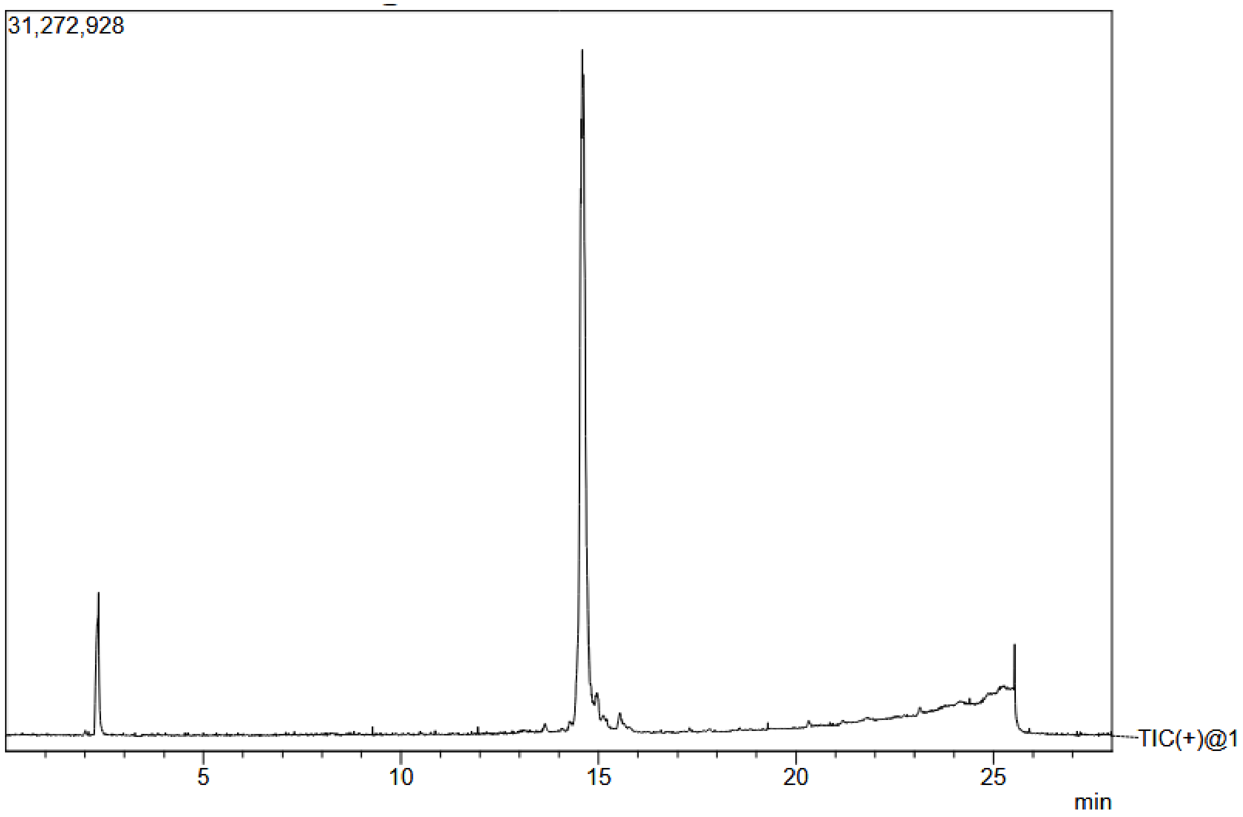

Figure S1: Total ion chromatograph of the raw GG-353 sample as received.

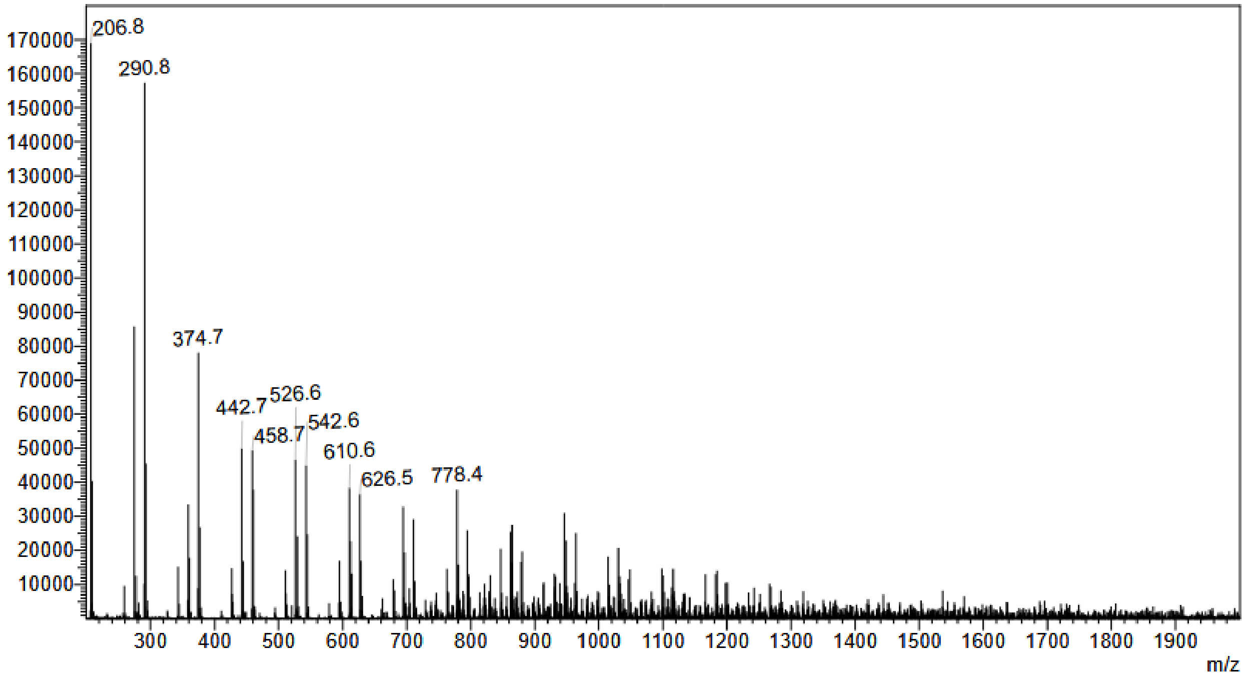

Figure S2: Extracted mass spectrum for the peak detected at t = 2.329 mins in Figure S1.

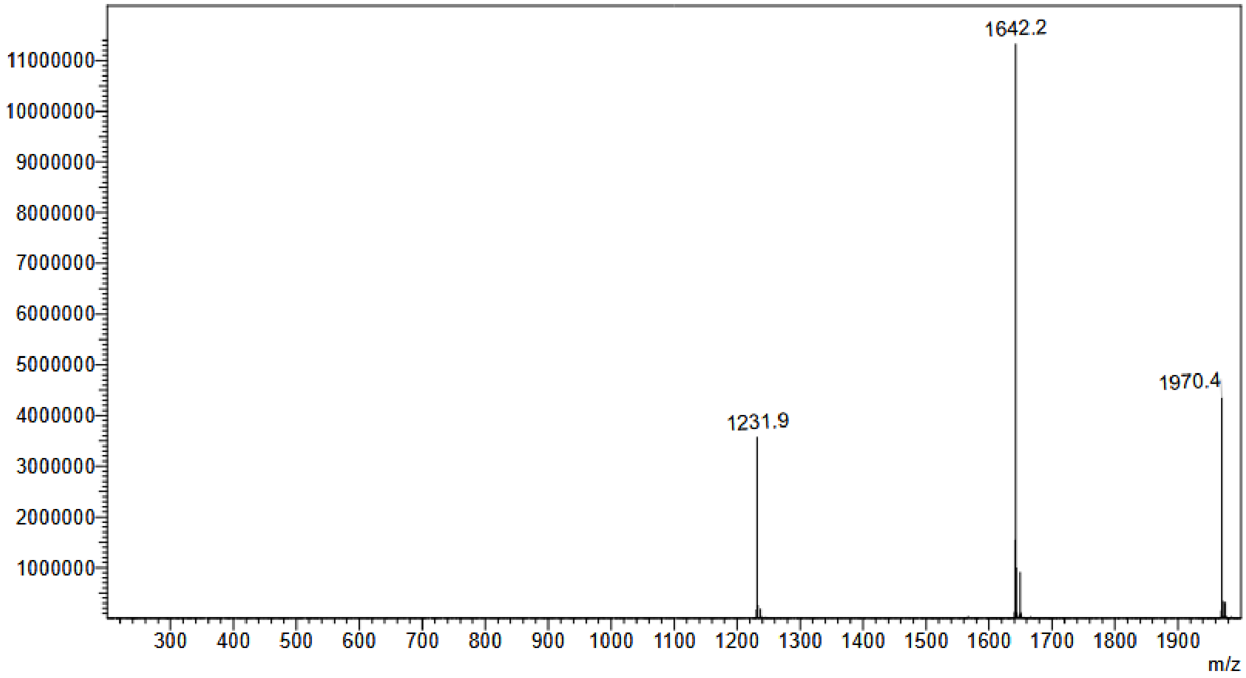

Figure S3: Extracted mass spectrum for the peak detected at t = 14.578 mins in Figure S1.

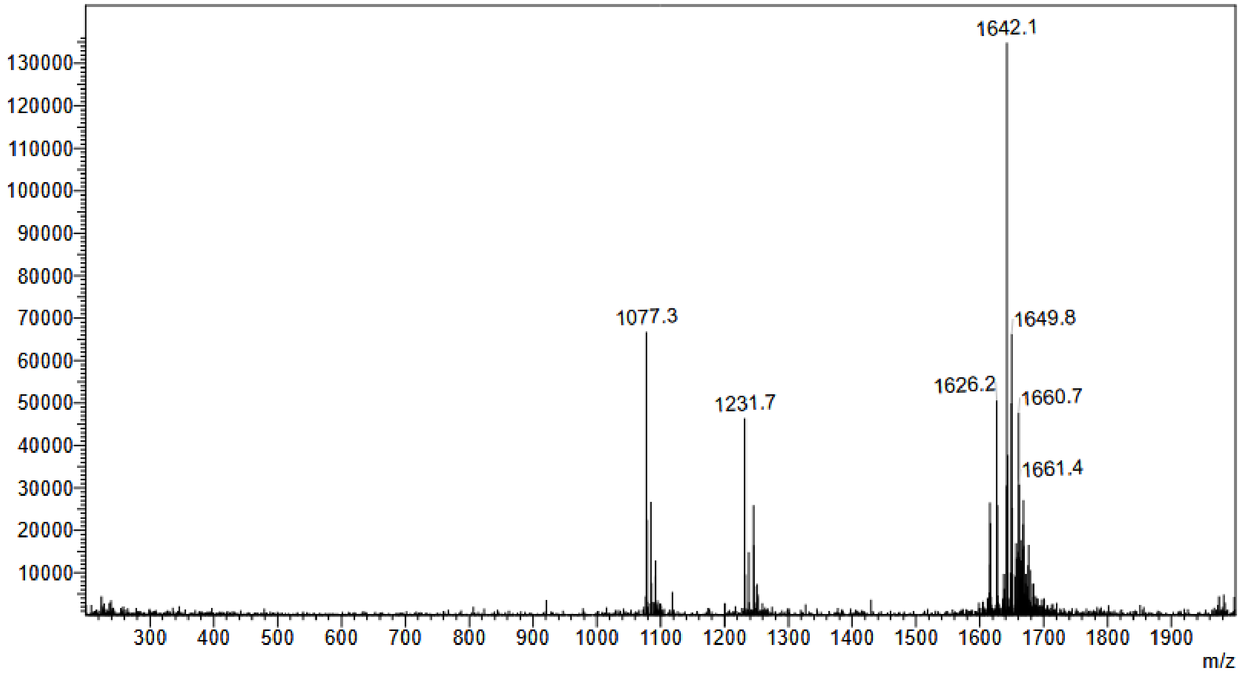

Figure S4: Extracted mass spectrum for the peak detected at t = 14.959 mins in Figure S1.

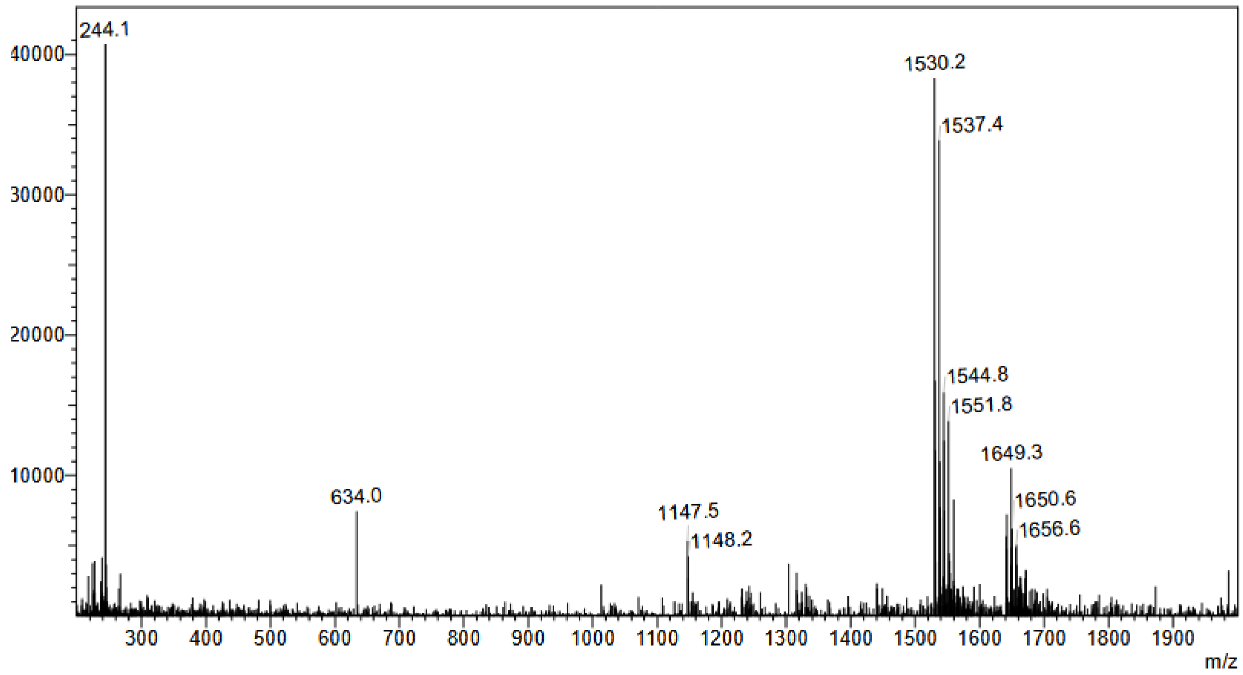

Figure S5: Extracted mass spectrum for the peak detected at t = 15.576 mins in Figure S1.

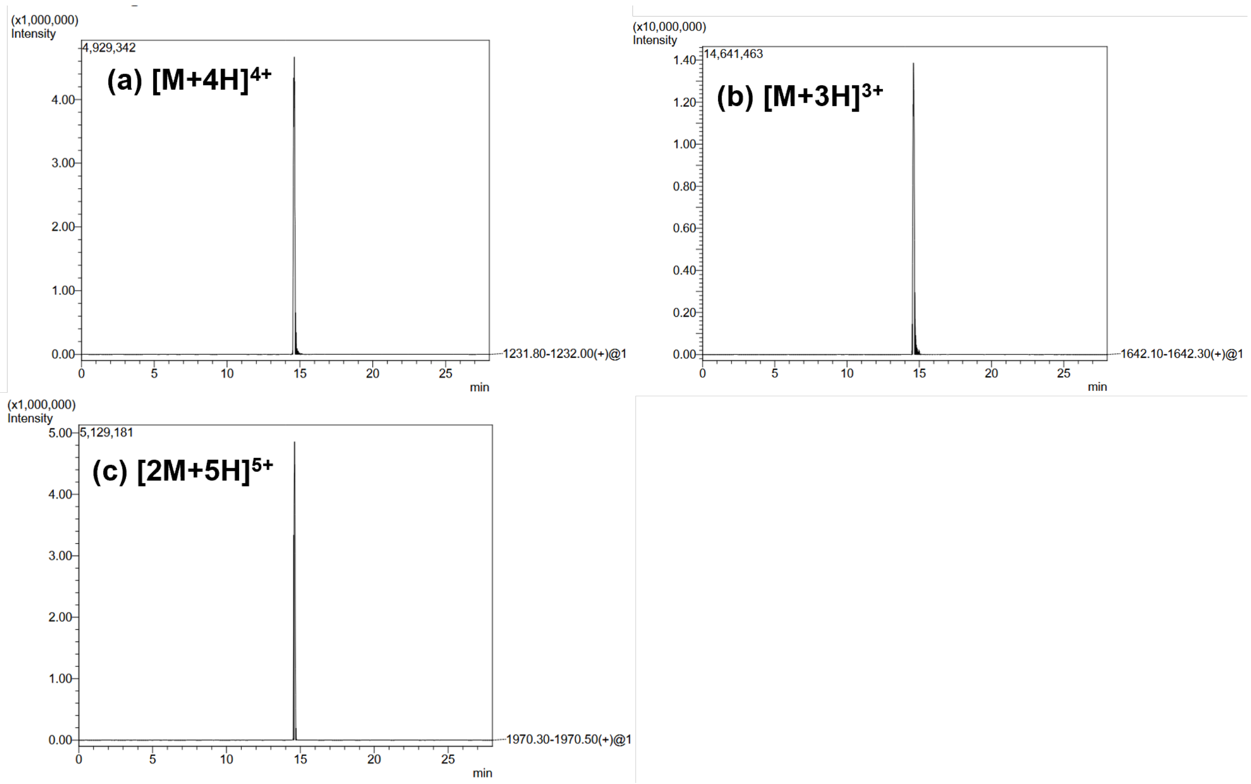

Figure S6: Extracted ion chromatographs for the intact peptide (M) at m/z values of (a) 1231.90±0.1 $[M'+4H]^{4+}$, (b) 1642.20±0.1 $[M'+3H]^{3+}$, and (c) 1970.40±0.1 $[2M'+5H]^{5+}$.

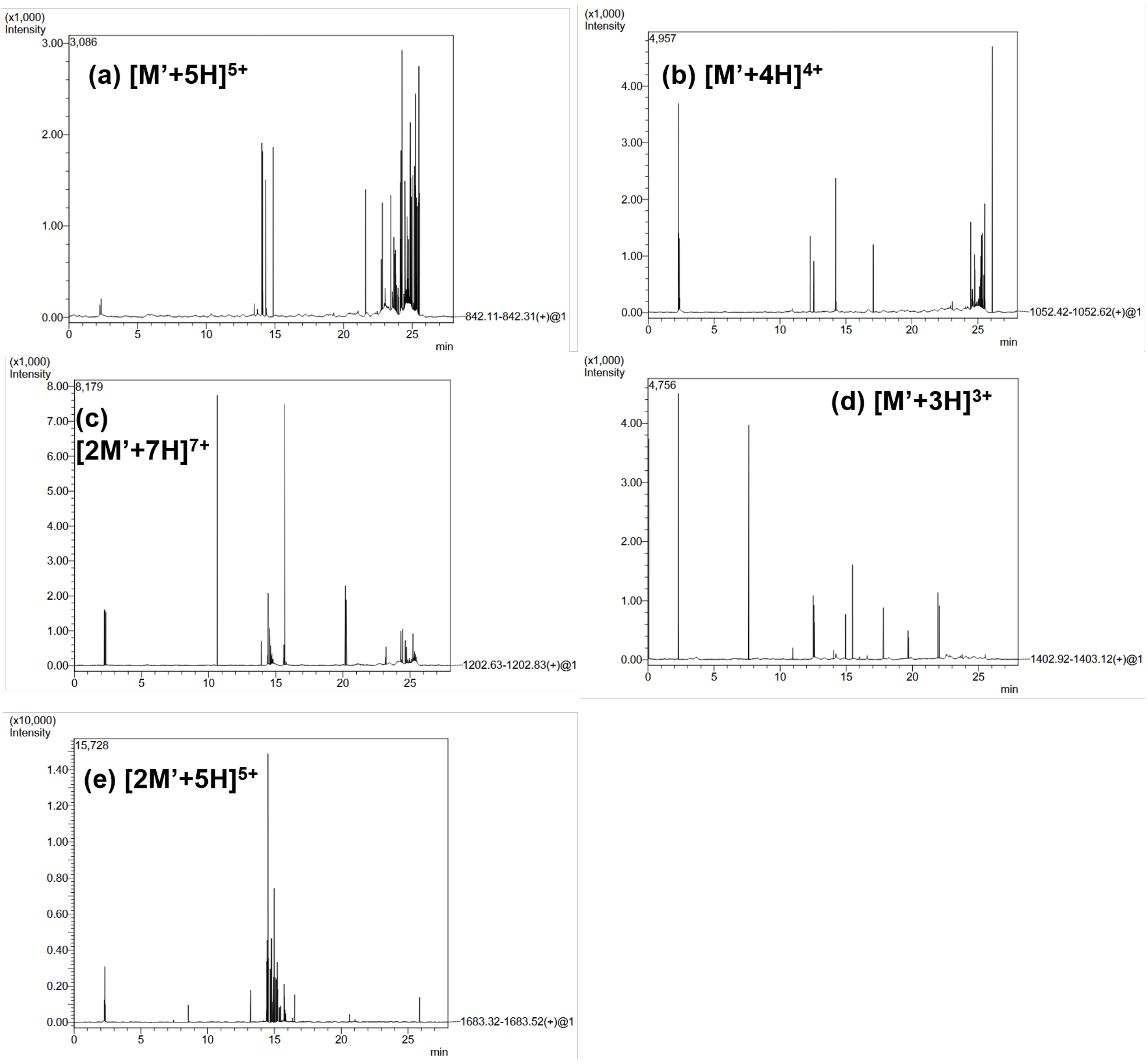

Figure S7: Extracted ion chromatographs for the potential deconjugated peptide (M’) at m/z values of (a) 842.21±0.1 $[M'+5H]^{5+}$, (b) 1052.52±0.1 $[M'+4H]^{4+}$, (c) 1202.73±0.1 $[2M'+7H]^{7+}$, (d) 1403.02±0.1 $[M'+3H]^{3+}$, and (e) 1683.42±0.1 $[2M'+5H]^{5+}$.

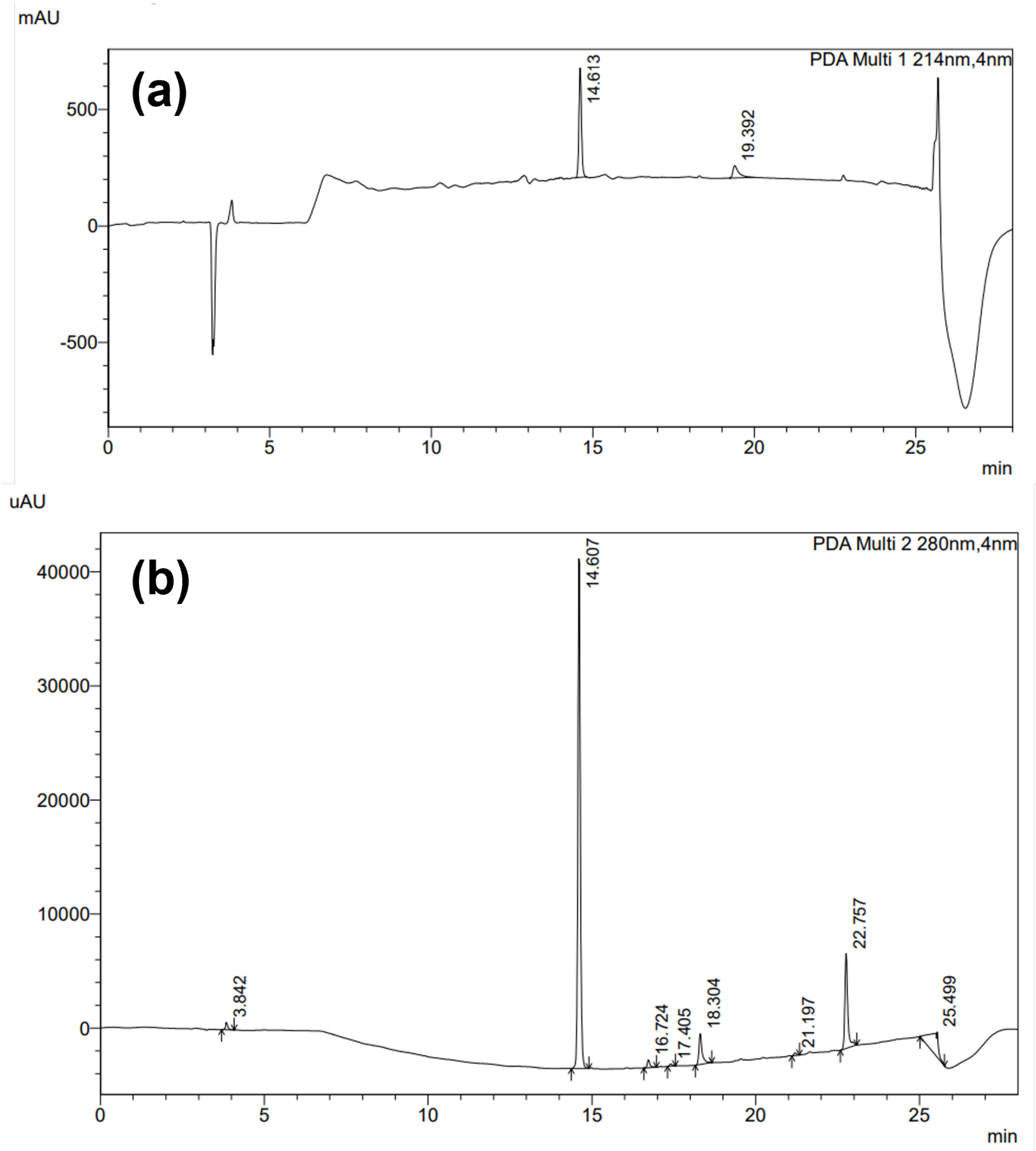

Figure S8: (a) 214 nm and (b) 280 nm chromatograms for the GG-353 raw material.

S2. Seed preparation protocol

The seed stock for microseed matrix screening experiments was created as follows:

1. The drop containing crystals was opened with a scalpel and 2 µL of precipitant solution was pipetted from the reservoir to the drop.
2. The drop was aspirated and dispensed multiple times to break up any polycrystalline clumps.
3. 2 µL of crystal suspension was aspirated and added to a microcentrifuge tube containing a PTFE seed bead (Hampton Research) and 50 µL of precipitant solution, which was kept in a beaker of ice.
4. Steps 1 through 3 were repeated until no crystalline material remained in the drop.
5. Steps 1 through 4 were repeated for all wells containing crystals, with the crystal suspensions being combined into a single microcentrifuge tube.
6. The crystals were crushed via vortex mixing of the resulting crystal suspension for 4 bouts of 30 seconds to generate the seed stock, and cooled on ice for 30 seconds between bouts.

A visual demonstration of the protocol is shown in Figure S9.

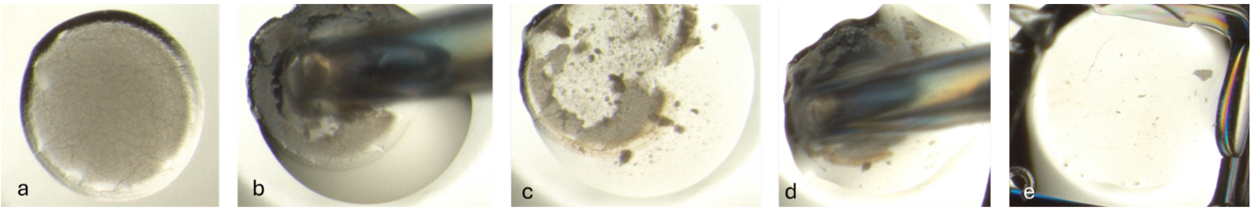

Figure S9: The process of generating seed stock from a crystalline drop. (a) A drop showing a polycrystalline mass. (b) A small volume of reservoir solution is added to the drop and mixed (c) The polycrystalline mass is crushed and the same volume of crystal suspension is withdrawn to the seed bead tube (d) another small volume of reservoir solution is added (e) the process is repeated until little-to-no crystalline material remains in the drop.

S3. Crystal images

Images of the crystal obtained during microseed matrix screening experiments are divided into three figures; those of identical habit to unseeded screens (Figure S10), those of a different habit (S11), and those observed in the absence of seeds (Figure S12).

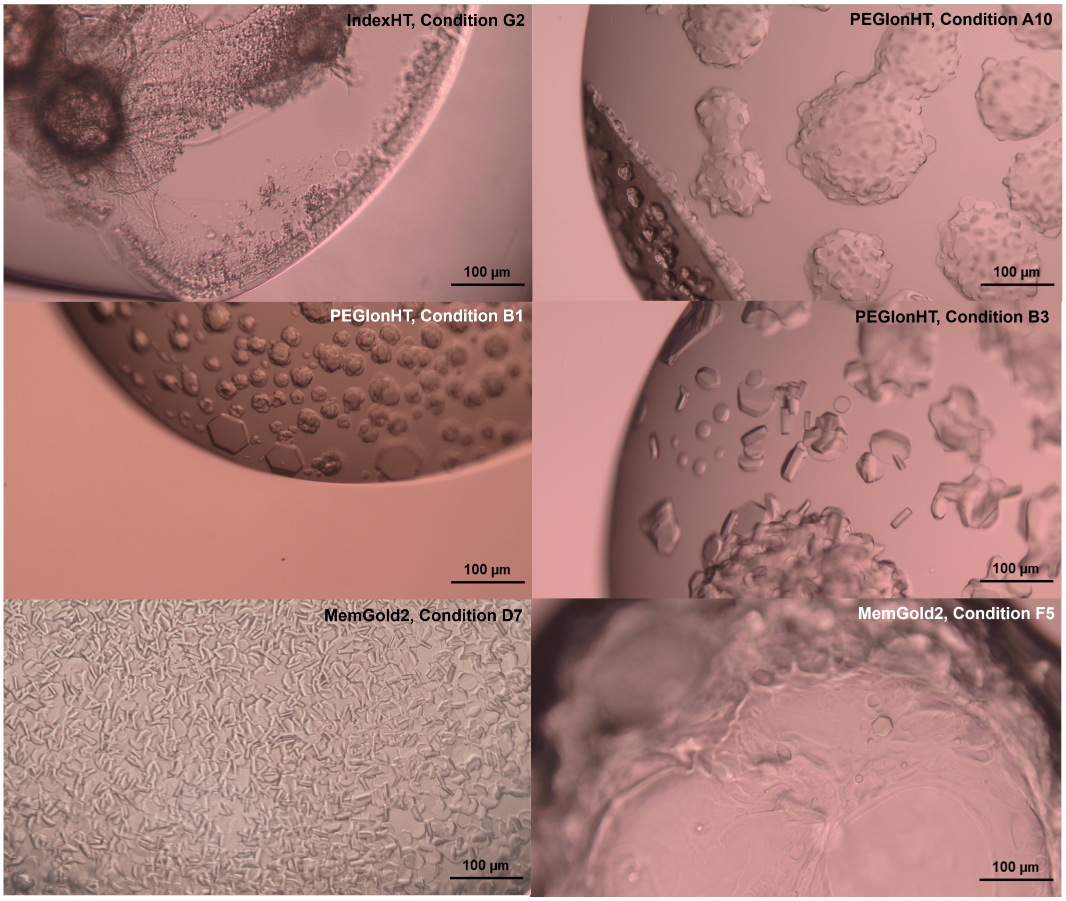

Figure S10: Microscope images of crystals obtained via MMS which were of similar habits to those obtained from previous optimisation attempts. Arrows are to guide the eye.

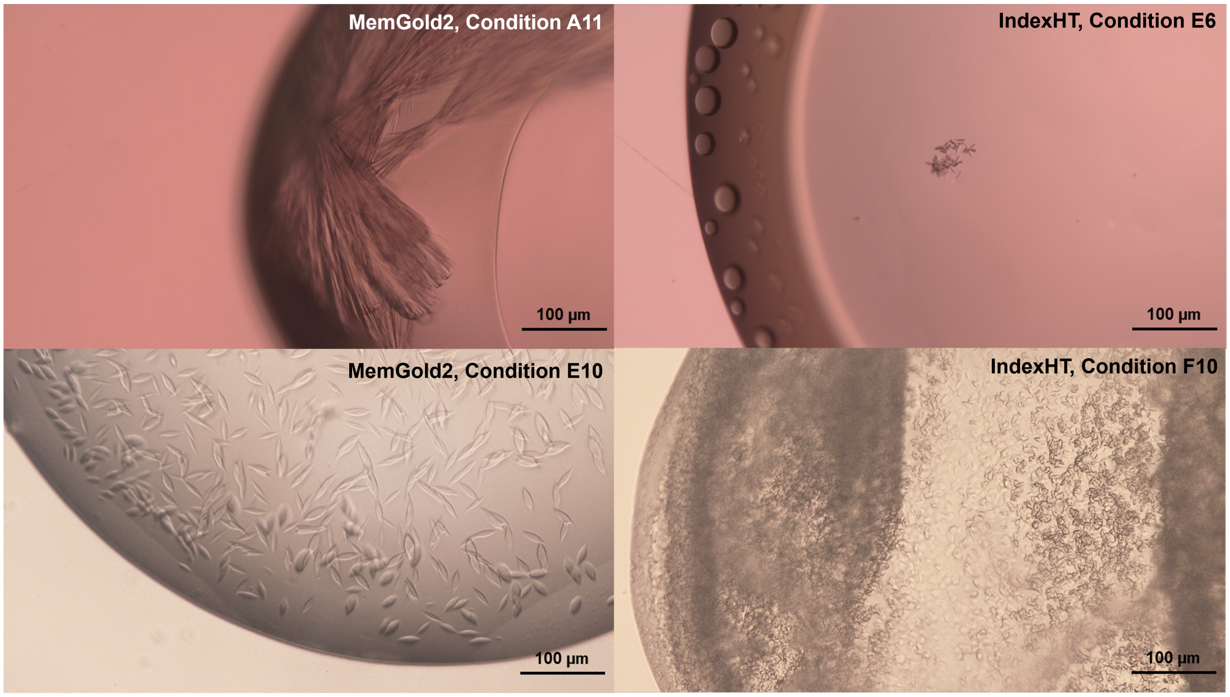

Figure S11: Microscope images of crystals obtained via MMS which were of different habits to those obtained from previous optimisation attempts.

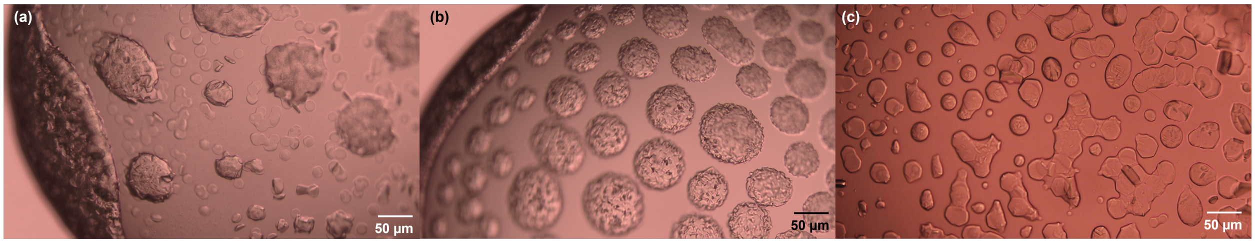

Figure S12: Unseeded hits uncovered during microseed matrix screening experiments. (a) Index condition G6. (b) Index condition F10 (Reservoir conditions: 0.1 M BIS-TRIS pH 5.5, 0.2M NaCl, 25% w/v PEG 3350). (c) PEGIon condition H10 (Reservoir conditions: 0.2 M NaBr, 20% w/v PEG 3350)

S4. Per-shell data reduction statistics

Table S2: Per-shell crystallographic statistics for GG-353 crystals after data reduction, as reported by AIMLESS.

d_{max}	d_{min}	No. reflections	No. unique reflections	Completeness	R_{pim}	Multiplicity	Mean I/σ(I)	$CC_{1/2}$
64.66	8.704	249	62	99.4	0.045	4.0	9.8	0.997
8.704	6.155	485	86	100.0	0.059	5.6	11.5	0.985
6.155	5.032	735	118	100.0	0.052	6.2	11.8	0.986

d_{max}	d_{min}	No. reflections	No. unique reflections	Completeness	R_{pim}	Multiplicity	Mean $I/\sigma(I)$	$CC_{1/2}$
5.032	4.356	807	127	100.0	0.044	6.4	12.1	0.988
4.356	3.895	808	132	100.0	0.045	6.1	11.9	0.989
3.895	3.556	1056	168	100.0	0.052	6.3	11.5	0.986
3.556	3.292	1008	162	100.0	0.057	6.2	9.9	0.980
3.292	3.079	1081	172	100.0	0.064	6.3	8.0	0.991
3.079	2.904	1105	182	99.4	0.077	6.1	7.1	0.956
2.904	2.754	1077	194	98.6	0.085	5.6	6.6	0.974
2.754	2.626	1229	210	100.0	0.099	5.9	6.3	0.958
2.626	2.514	1413	236	100.0	0.125	6.0	4.9	0.960
2.514	2.415	1219	207	100.0	0.121	5.9	4.8	0.964
2.415	2.327	1235	210	100.0	0.158	5.9	4.0	0.947
2.327	2.249	1405	241	100.0	0.187	5.8	3.9	0.933
2.249	2.178	1549	257	100.0	0.175	6.0	4.6	0.936
2.178	2.112	1667	265	100.0	0.198	6.3	3.7	0.888
2.112	2.053	1590	249	100.0	0.272	6.4	2.9	0.817
2.053	1.998	1774	270	100.0	0.305	6.6	2.4	0.750
1.998	1.947	1701	261	100.0	0.448	6.5	1.6	0.676
1.947	1.901	1669	260	99.2	0.502	6.4	1.6	0.585
1.901	1.857	1806	298	100.0	0.521	6.1	2.0	0.645
1.857	1.816	1947	307	100.0	0.713	6.3	1.4	0.493
1.816	1.778	1985	303	100.0	0.788	6.6	1.2	0.417
1.778	1.742	1926	288	100.0	0.827	6.7	1.1	0.460
1.742	1.708	2089	309	100.0	1.059	6.8	0.9	0.267
1.708	1.676	2069	307	100.0	1.648	6.7	0.6	0.073
1.676	1.646	2023	302	100.0	1.569	6.7	0.6	0.066
1.617	1.590	2214	353	100.0	1.391	6.3	0.7	0.173

S5. Ramachandran analysis

Ramachandran plots for the GG-353 crystal structure are given in Figure S13.

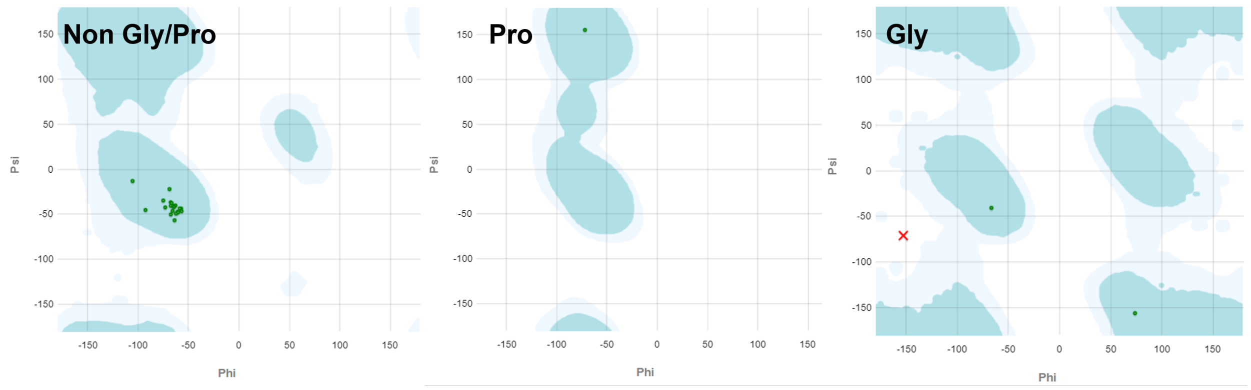

Figure S13: Ramachandran analysis of the residues of GG-353 as per Iris.
